# Effects of swine manure and straw biochars on fluorine adsorption-desorption in soils

**DOI:** 10.1371/journal.pone.0302937

**Published:** 2024-05-16

**Authors:** Jiatao Cui, Mengyu Zhang, Meng Mi, Yaming Zhao, Zewen Jin, Ming Hung Wong, Shengdao Shan, Lifeng Ping

**Affiliations:** 1 Key Laboratory of Recycling and Eco-Treatment of Waste Biomass of Zhejiang Province, Zhejiang University of Science and Technology, Hangzhou, PR China; 2 Consortium on Health, Environment, Education, and Research (CHEER), Department of Science and Environmental Studies, The Education University of Hong Kong, Hong Kong SAR, Hong Kong, China; Jazan University, SAUDI ARABIA

## Abstract

With increasing global awareness of soil health, attention must be paid to fluorine exposure in soils, which poses a threat to human health. Therefore, this study aimed to study the fluorine adsorption characteristics of swine manure and straw biochars and their impact on fluorine adsorption-desorption in soil with batch experiments. The biochar samples originated from high-temperature anaerobic cracking of swine manure (350°C, 500°C, and 650°C) and straw (500°C). Results indicated that the adsorption of soil fluorine reached adsorption equilibrium at around 4 h after the mixing of swine manure and straw biochar. Fluorine adsorption kinetics using these biochars conformed to the quasi-two-stage kinetic model. The fluorine adsorption kinetics for biochar-treated soils conformed to the double-constant equation and the Elovich equation, and the soil treated with straw biochar showed the fastest fluorine adsorption rate. The adsorption isotherms of fluorine for biochars and biochar-treated soils could be fitted by the isothermal adsorption model of Langmuir and Freundlich. The maximal equilibrium quantity of fluorine was 73.66 mg/g for swine manure biochar. The soil, adding with 2% of swine manure biochar achieved with showed at 650°C had the smallest adsorption. This study also shows that the adsorption of fluorine by biochar gradually decreased with the increase of pH. Comparing with other factors, the mixture pH with biochars added had a significant effect on fluorine adsorption. The decreased fluorine adsorption capacities for soils treated with swine manure and straw biochars were closely related to the increased pH in soils after adding biochars. Considering the fluorine threat in soil, this study provides a theoretical basis for the application of biochars on soil fluorine adsorption.

## Introduction

Fluorine (F) is the most electronegative halogen and chemically reactive element. It is ubiquitously present in rocks, soil, air, and water, predominantly in fluorine [1]. Fluorine is a vital trace element. With minute amounts (0.05–0.07 mg/L), enhancing the hardness of tooth enamel and effectively preventing dental caries. However, excessive fluorine intake can lead to dental fluorosis and osteofluorosis, and can adversely affecting the immune system, kidneys, and gastrointestinal tract [2, 3]. Human exposure to fluorine primarily occurs through food and drinking water, and soils become a significant source of fluorine as soils can affect the groundwater and food in many regions.

As modern industrial and agricultural activities continue to expand, the soil fluorine contents are rising. Phosphate fertilizer plants, aluminum smelters, brick and tile factories, along with the usage of fluorine-containing pesticides contribute to increased fluorine levels in the soil around these facilities. These, in turn, affect the fluorine contents in the plants and groundwaters of these areas [4].

Fluorine is highly biologically active, even in areas with low levels of fluorine contamination. Plants can enrich fluorine in the environment, and threaten wildlife and humans through the food chain [5]. For example, crops grown on the ferroaluminum soils in the south of China are highly fluoridated, resulting in a very high risk of fluorosis. In addition, many areas including areas in Zhejiang province, Longxi in Fujian province, Meizhou and Huiyang in Guangdong province have become hot springs-type fluorosis areas due to localized fluoride-rich environments. Hence, the migrations and transformations of fluorine in soils become an important issue that cannot be ignored [6].

Biochars, novel environmental-friend functional materials, have distinctive properties such as large specific surface areas, pore-rich structures with functional groups, and excellent biochemical stabilities [7–9]. The modified biochars usually show excellent adsorption capacities [10]. Nowadays, biochars are often derived from agricultural waste through thermal pyrolysis under limited oxygen conditions and have been widely used in soil improvement and remediation [11–13]. Consequently, considering the threatens of fluorine exposure risks to human lives via fluorine contaminated soils, the studies for the impact of environmental-friend biochars on fluorine adsorption-desorption characteristics in high-fluorine soils become crucial, provide potential solutions for the soil fluorine removal.

Now, various techniques have been employed for defluorination of water or soil, including phytoremediation [14], electrodialysis [15], ion exchange processes [16], electrocoagulation [17], and adsorption. Among these methods, adsorption is widely recognized as the most sustainable approach from both technical and economic perspectives due to its high removal efficiencies, ease of operations, low costs, and potential for recovery and regeneration of adsorbent materials [18]. Among different types of adsorbent materials, biochar-based adsorbent materials derived from pyrolyzed biomass waste draw researcher attentions as their sources are environmental sustainable.

Gao et al. [19] used charcoal and bamboo charcoal as amendment to adsorb fluoride in tea garden soil, and showed fluoride removal capacities of 1.42 mg/g and 1.65 mg/g, respectively. Sun et al. [20] found that the hydrothermal modified fly ash showed maximum fluorine adsorption capacity of 47.35 mg/g when employed to remove fluorine in waste water under alkaline conditions. Fluorine adsorption capacities of 6.58 mg/g and 3.42 mg/g were achieved with biochar produced from bamboo and rice husks, respectively [21], and Sadhu et al. [22] showed fluoride removal capacity of 9.5 mg/g for groundwater samples using biochar produced from watermelon rind.

In addition, the pyrolysis temperatures for biochar productions play a crucial role in determining the costs and adsorption efficiencies. Some studies [23, 24] have shown that a significant decrease in both porosities and surface areas of biochars produced from marine macroalgae residues and almond/nut shells happened when the thermal pyrolysis temperatures went to 800°C from 400°C. With the pyrolysis temperature increasing, the softening, melting, fusing, and carbonization of the biochar structures will happen and result to pore widening, coalescence of adjacent pores, and partial pore blockage, which decrease the biochar porosities and surface areas [25]. Conversely, relatively low pyrolysis temperatures for biochar productions can lead to incomplete carbonization of the biomass, resulting in reduced loss of volatiles and small surface areas [26]. Therefore, careful optimizations of the pyrolysis temperatures are necessary to strike a balance between cost-effectiveness and the desired adsorption performances.

Biochars have been studied for the fluorine adsorption, but the biochars from agriculture waste especially from swine manure which exists in China with billion tons are not studied for the fluorine adsorption. Here, fluorine adsorption-desorption behaviors using swine manure and straw biochars were studied. A series of batch experiments to examine the sorption and desorption properties of fluorine in soil were carried out, with biochars achieved with different pyrolysis temperatures. The impact of various factors such as reaction duration time, initial fluorine concentration and mixture pH were also studied. Furthermore, the kinetics and equilibrium isotherms were applied to gain insight into the fluorine adsorption mechanism using biochars [27, 28].

This study aims to understand how biochar influences fluorine dynamic adsorption-desorption behaviors in soil, and the findings from this work will provide essential data for the potential removal of fluorine migration and associated risks, which now become big concern of the soil safeties.

## Materials and methods

### Materials

#### Biochar

The raw material of biochar used in the experiment was swine manure from Shunkang animal husbandry in Kaihua, Zhejiang Province. The swine manure and straw biochars were prepared by anaerobic carbonization by Jinhua Jinguo Company, Zhejiang Province. The biochar specimens subjected to testing originated from high-temperature anaerobic cracking of swine manure (350°C, 500°C, and 650°C) and straw (500°C), which were named as PBC350, PBC500, PBC650, and RBC500, respectively. their fundamental physicochemical characteristics (on a dry weight basis) are presented in Table 1, while the specific surface areas and the pore structures were determined via BET-specific surface areas as shown in Table 2.

**Table 1:** Physicochemical properties of biochars.

Sample	pH	Ash (%)	TP (g/kg)	TK (g/kg)	TF (g/kg)
**PBC350**	7.61	16.94	11.31	17.60	0.25
**PBC500**	9.07	47.35	66.67	25.80	0.16
**PBC650**	9.32	48.52	62.78	24.30	0.15
**RBC500**	7.32	18.21	11.14	17.74	0.13

Note: TP: total phosphorus; TK: total potassium; TF: total fluorine

**Table 2:** BET characteristics of biochars.

Sample	SSA (m^2^/g)	PV (cm^3^/g)	PD (nm)
**PBC350**	14.63	0.0297	10.76
**PBC500**	16.30	0.0266	9.63
**PBC650**	22.69	0.0364	8.26
**RBC500**	13.47	0.0436	14.61

Note: SSA represent specific surface area; PV represent average pore size; PD represent Total pore volume.

#### Test soil samples

The soil samples were taken from HaiShun Farm, Chihuai Town, Kaihua County, Quzhou City, Zhejiang Province (118°58’E, 29°10’N). They were yellow loams and one of them was also served as the experiment’s control soil (CK). The control soil exhibited the following baseline physicochemical characteristics: a pH value of 4.01(soil to water ratio at 1:5), organic matter content of 4.92 g/kg [29], with compositions comprising 27% sand grains, 45% silt grains, and 28% clay grains [30], along with 1421.25 mg/kg of total fluorine [31] and 9.12 mg/kg of water-soluble fluorine [32].

The tested soil samples encompassed variations with adding 0.25%, 0.5%, 1%, and 2% biochars to the control soil samples. The soil samples mixed with swine manure biochars, obtained at pyrolysis temperatures of 350°C, 500°C, and 650°C, were denoted as A group (A1-A4), B group (B1-B4), and C group (C1-C4) samples, respectively. Additionally, the soil samples mixed with straw biochars obtained at 500°C were designated as D group (D1-D4) samples.

These distinctions in soil composition could show us a comprehensive study on the impact of biochars, originating from different sources and pyrolysis temperatures, on the physicochemical characteristics of the soils.

### Experimental methods

#### Adsorption kinetics experiments

The batch experiments were conducted at room temperature (25±1°C) using a mechanical shaker. The samples were placed in 50 mL centrifuge tubes and shaken at 180 rpm. Each tube contained 0.1 g of biochars or 1.0 g of soil samples mixed with different ratios of biochars, along with 20 mL of 100 mg/L fluorine solutions. The pH of reaction solutions were varied from 4 to 8 by adding 1 M NaOH or 1 M HCl. The shaking processes lasted for 1, 5, 10, 15, 20, 30, 45, 60, 120, 240, 360, 720, and 1440 min. Each experiment consisted of three replicates, and there were also blank samples without any soil samples or biochars.

The amounts of fluorine in biochar and soil samples were calculated using Eq (1):

$$q_{t}=\frac{(C_{0}-C_{t})\times V}{W}$$ (1)

The variables *q*_*t*_ (expressed in mg/kg) represent the quantities of fluorine that have been adsorbed by the experimental samples at equilibrium during a given time. *C*_0_ and *C*_*t*_ (given in mg/L) represented the fluorine concentrations in the solutions before the start of the adsorption processes and after certain incubation times for adsorption processes (t), respectively. V (given in mL) represented the volumes of the solutions added, while W (given in g) denoted the weights of the soil samples studied.

#### Adsorption isotherms experiments

These experiments were also conducted at room temperature (25±1°C) using a mechanical shaker. The samples were placed in 50 mL centrifuge tubes and shaken at 180 rpm. Each tube contained 0.1 g of biochars or 1.0 g of soil samples mixed with biochars, and 20 mL solutions with different fluorine concentrations of 5, 10, 25, 50, 100, 300, 500, 800, and 1,000 mg/L were added. The shaking processes lasted for 240 min to reach equilibrium. After standing for 2 h, the solutions were centrifuged at 4,000 rpm for 5 min, and the resulting supernatants were used for the fluorine concentration measurements.

The equations provided below were utilized to determine the fluorine adsorption capacities (referred to as *q*_*e*_) and the fluorine elimination rates (*R*) of samples. These equations took into account the disparities between the total amounts of fluorine present and the amounts that remained in the solution.

$$q_{e}=\frac{v(C_{0}-C_{e})}{W}$$ (2)

$$R=\frac{C_{0}-C_{e}}{C_{0}}\times 100$$ (3)

The variables *q*_*e*_ (mg/kg) represented the quantities of fluorine that have been adsorbed at equilibrium. *C*_0_ represented the initial concentration of F^−^ (mg/L), and *C*_*e*_ represented the equilibrium concentration of F^−^ (mg/L).

#### Desorption experiments

Desorption experiments were carried out immediately after the completion of the adsorption tests. The processes involved removing supernatant solutions to get the precipitated samples within the tubes. Each tube was then supplied with approximately 25 mL of deionized water and subjected to agitation using a mechanical shaker at a speed of 180 rpm for 4 h duration at room temperature (25±1°C). The amounts of fluorine desorbed were then calculated using the following equation, represented as Eq (4)

$$q_{des}=\{c_{e}^{des}(25+w_{2}-w_{1})-c_{e}(w_{2}-w_{1})\}\times 1000$$ (4)

In the given equation, the term $c_{e}^{des}$ (expressed in mg/L) represented the fluorine concentrations in the equilibrium solutions after the desorption processes. The variable w_1_ (given in g) denoted the total weights of the tube and soil samples, while w_2_ (given in g) represented the combined weights of the tube, soil samples, and the remaining solutions after adsorption. The calculations of the fluorine desorption rate (*R*_*des*_) were caculated using the equation stated as Eq (5)

$$R_{des}=\frac{q_{des}}{q_{e}}\times 100$$ (5)

$$Net\;adsorption\;amount\;(mg\cdot kg^{‐1})\;=\;q_{e}-q_{des}$$ (6)

### Analytical detection method

The fluorine ion concentrations in the solution were measured using the fluorine electrode method (PXSJ-216F, LeiMagnet, China). The biochar BET-specific surface areas and pore structures were analyzed using surface and pore size analyzer (ASAP 2020 Plus HD88). Additionally, infrared spectral analysis was conducted using a Fourier infrared spectrometer (ThermoFisher Nicolet ls50).

Origin2021 was used for statistically analyzations of the experimental datas.

## Results and discussions

### Adsorption kinetics of fluorine

#### Adsorption kinetics of fluorine using biochars

**Fig 1:**
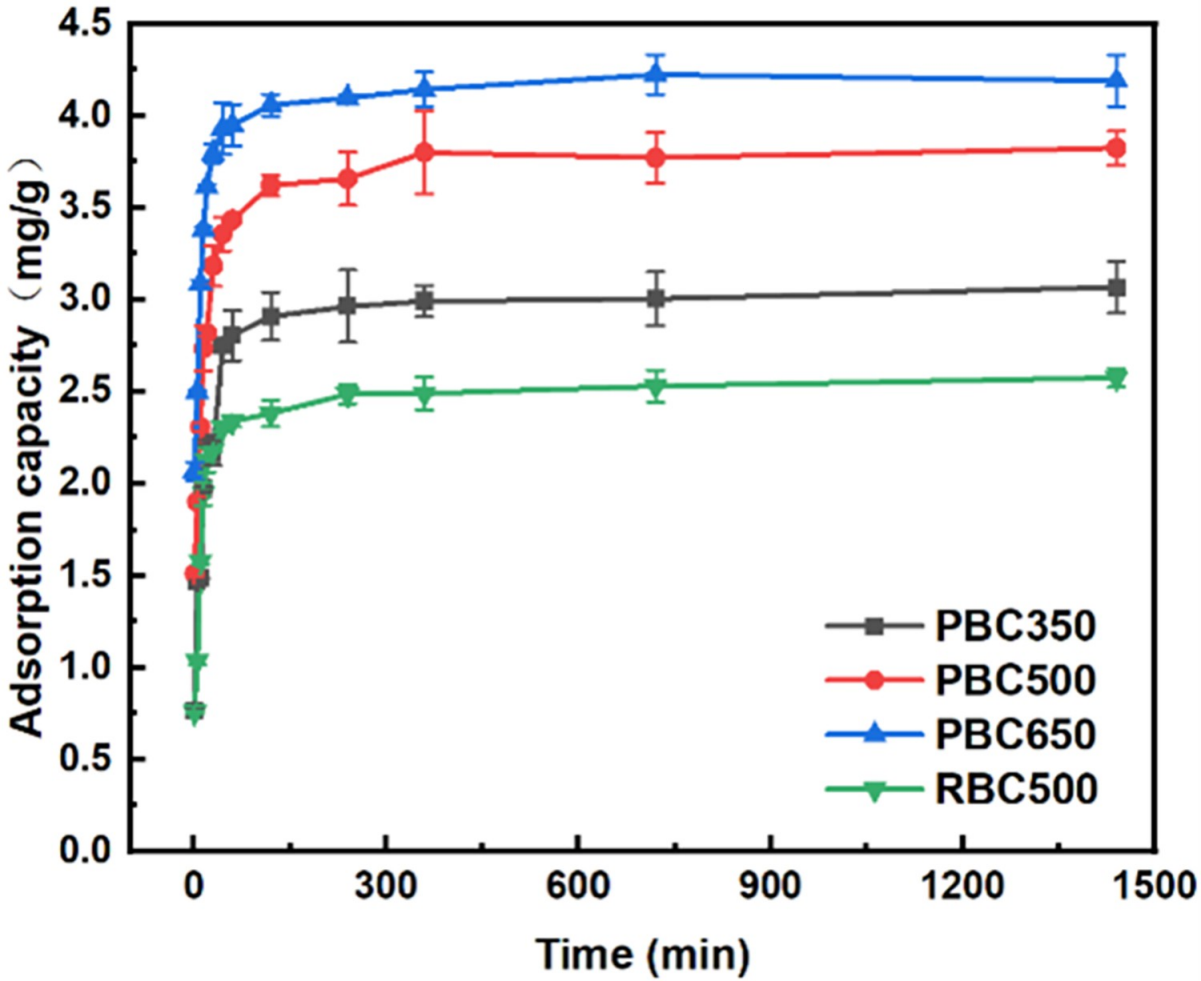
Adsorption kinetic curves with different types of biochars.

The adsorption kinetics of fluorine using different types of biochars varied, and the adsorption kinetics curves were shown in Fig 1. At room temperature, with an initial concentration of 100 mg/L fluorine, the fluorine adsorption efficiencies and amounts for all the biochar samples increased as the incubation times lengthened. Initially, fluorine adsorption occurred rapidly within the first 240 min. After 240 min, there were no significant changes for fluorine concentrations in solutions, indicating the attainment of adsorption equilibrium. At 1440 min, the maximum fluorine adsorption amounts were 3.1 mg/g for PBC350, 3.8 mg/g for PBC500, and 4.3 mg/g for PBC650 of swine manure biochars, while the fluorine adsorption amount was 2.6 mg/g for straw biochar RBC500. Notably, swine manure biochar obtained at 650°C showed the highest effectiveness for fluorine adsorption.

The adsorption rates of fluorine by all swine manure and straw biochars exhibited first rising and then decreasing trend, with equilibrium reached at 240 minutes. The kinetic curves revealed a clear two-stage adsorption process: fast adsorption rate in the initial two hours and slower adsorption rate in the subsequent two hours. Approximately 88%-92% of the maximum adsorption capacities were achieved in the first two hours, as it was reported that fluorine the adsorptions by biochars were usually caused by both physical adsorptions and chemical adsorptions [33]. Active sites on the biochar surface played a crucial role initially, leading to a faster adsorption rate of fluorine. With the extensions of incubation times, the active sites for fluorine binding were nearly saturated, fluorine needed diffusion into the inner surfaces and pores for adsorptions which lead to slower fluorine adsorption rates [13]. At equilibrium, the fluorine adsorption capacities for the biochars followed this order: swine manure biochar (650°C) > swine manure biochar (500°C) > swine manure biochar (350°C) > straw biochar (500°C).

To further study the fluorine adsorption kinetics using various biochars, the kinetic adsorption results were fitted by Lagergren quasi-primary kinetic model, quasi-secondary kinetic model, double constant equation, and Elovich equation, among which the quasi-primary kinetic model and quasi-secondary kinetic model showed good fitting effects (R^2^>0.7), and Table 3 presented the corresponding fitted parameters. Based on fitting data, it was found that the R^2^ for the quasi-secondary kinetic equation were significantly higher than R^2^ for the quasi-primary kinetic equation in all experiments. The better fitting using quasi-secondary kinetic equation indicated that the fluorine adsorption by swine manure and straw biochars were mainly based on chemosorption. The adsorption mechanism involved a comprehensive effect of surface adsorption and fluorine diffusion within the particles [34]. For using the quasi-secondary kinetic equation, the k₂ value of swine manure biochar (650°C) was the highest, suggesting that efficient adsorption happened at the pore channels and functional groups of swine manure biochar surfaces. Additionally, the fluorine in the solution swiftly occupied the pores of the biochars and their active adsorption sites, which could effectively promote the adsorption rate. Straw biochar (500°C) showed a higher k₂ value than that of swine manure biochar (350°C and 500°C), but it had a smaller equilibrium adsorption amount, which indicated that a rapid ligand-exchange adsorption reaction happened in the early stage, followed by slow diffusion and the formation of new minerals in the late stage for fluorine adsorption using straw biochar. In contrast, swine manure biochar exhibited more precipitation reactions for mineral formations, leading to higher fluorine adsorption capacities.

**Table 3:** Kinetic model parameters for the fluorine adsorption using biochars.

Sample	Pseudo first-order			Pseudo second-order		
	*q*_*e*_ (mg/g)	K_1_ (min^-1^)	R^2^	*q*_*e*_ (mg/g)	k_2_ (g·(mg·min)^−1^)	R^2^
**PBC350**	2.851	0.186	0.898	2.996	0.046	0.928
**PBC500**	3.487	0.226	0.704	3.643	0.060	0.853
**PBC650**	3.923	0.517	0.773	4.049	0.127	0.856
**RBC500**	2.427	0.246	0.902	2.552	0.072	0.952

Classical kinetic models (Eqs (7), (8), (9) and (10)) [35] were employed to fit the adsorption amount results of fluorine adsorption using biochars and soil samples at different reaction times.

$$Quasi‐primary\;kinetic\;equation:\;log(q_{e}-q_{t})=logq_{e}-\frac{k_{1}t}{2.303}$$ (7)

$$Quasi‐secondary\;kinetic\;equation:\;\frac{t}{q_{t}}=\frac{1}{k_{2}q_{e}^{2}}+\frac{t}{q_{e}}$$ (8)

$$Double\;constant\;equation:\;lnq_{t}=a+b\times ln_{t}$$ (9)

$$Elovich\;equation:\;q_{t}=a+b\times ln_{t}$$ (10)

Where k_1_ represented the quasi-primary reaction rate constant (min^-1^), k_2_ represented the quasi-secondary reaction rate constant (g·(mg·min)^−1^); the a-value represented the adsorption rate [36], and b-value represented the change of adsorption activation energy [37].

#### Adsorption kinetics of fluorine in biochar treated soils

**Fig 2:**
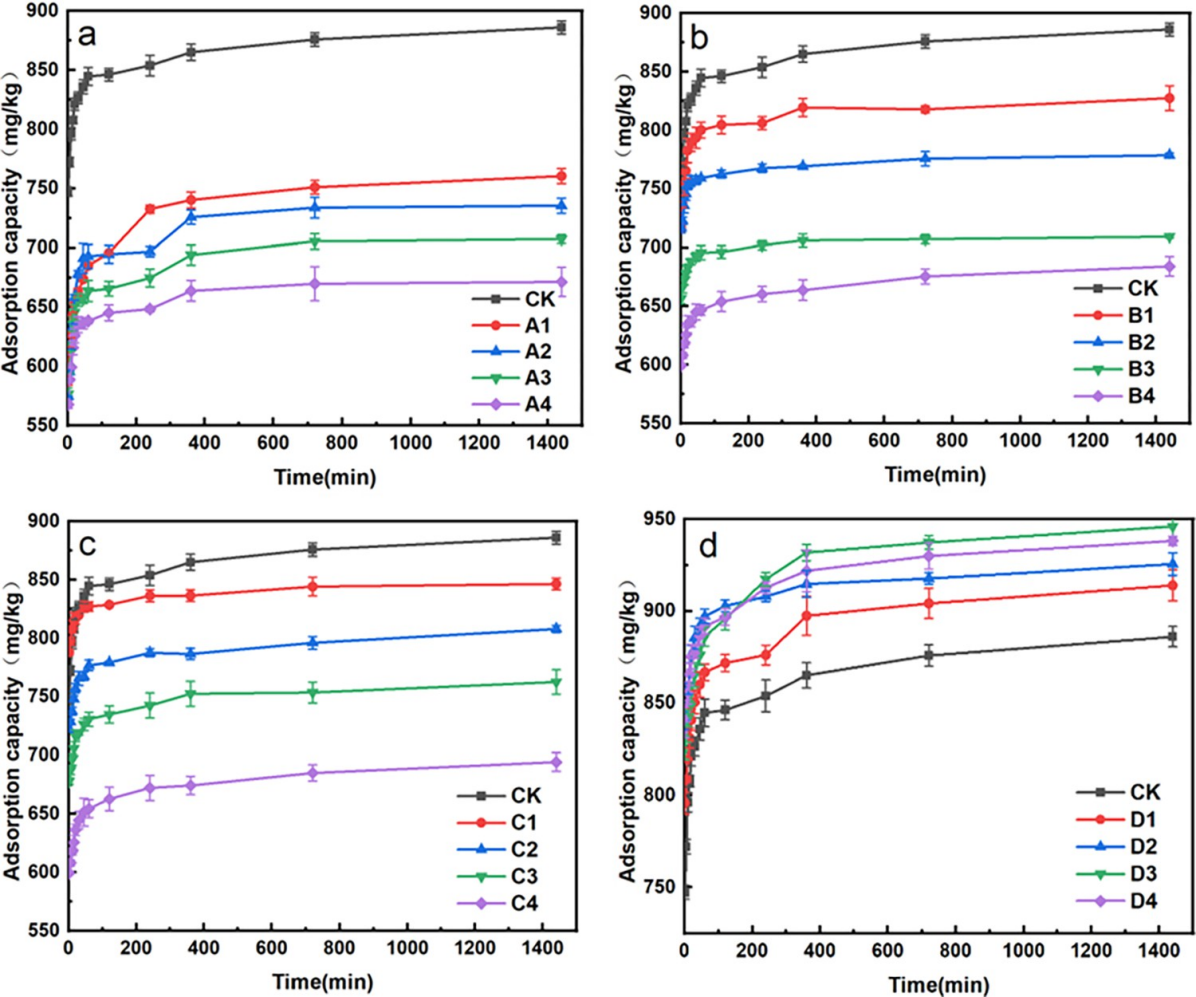
Fluorine kinetic adsorption curves using different soil samples. (Note: a, swine manure biochar (350°C)-treated soil; b, swine manure biochar (500°C)-treated soil; c, swine manure biochar (650°C)-treated soil; d, straw biochar (500°C)-treated soil).

The fluorine adsorption kinetic curves for different soil samples were illustrated in Fig 2. The fluorine adsorption amount showed positive correlations with adsorption time, showcasing distinct phases of fast and slow fluorine adsorption rates. All the treatment soil samples showed the same trends: gradual fluorine adsorption capacities increased with the extensions of incubation times. The fluorine adsorption capacities of all the samples showed rapid increased trends within initial 240 min, followed by gradual slowing down trends, and the relative equilibriums were reached after 1440 min.

It was found that the swine manure biochar treated soils displayed lower fluorine adsorption capacities than that using original soil. Moreover, as the amounts of biochar increased, the fluorine adsorption capacities in swine manure biochar-treated soil gradually decreased. Conversely, soil treated with straw biochar exhibited higher fluorine adsorption amounts than that of the original soil, with the fluorine adsorption capacities showed positive correlations with the amounts of biochar adding.

The quasi-primary kinetic model, quasi-secondary kinetic model, double constant equation, and Elovich equation [36] were employed to delve deeper into the kinetic properties of fluorine ion adsorption in biochar-amended soils. The double constant equation and Elovich equation both exhibited good nonlinear fitting coefficients (R^2^ > 0.90). Their fitting parameters were detailed in Table 4. Among the using of these four biochar types, the soil samples treated with straw biochar (500°C) displayed the highest a-value and b-value, indicating the fastest fluorine adsorption rates in the early adsorption stage and the best fluorine adsorption performances. In contrast, soil samples treated with swine manure biochar (350°C) showed the smallest a-value and the slowest adsorption rate. Soil samples treated with swine manure biochar (500°C) exhibited the smallest b-value, indicating relatively poor fluorine adsorption performance.

**Table 4:** Kinetic model parameters for the fluorine adsorption in biochar treated soils.

Sample	Double constant			Elovich		
	A	B	R^2^	a	b	R^2^
**CK**	6.649	0.0183	0.988	767.6	15.62	0.987
**A1**	6.493	0.0170	0.962	656.6	13.18	0.964
**A2**	6.433	0.0131	0.938	616.2	12.44	0.937
**A3**	6.420	0.0138	0.957	610.0	12.89	0.961
**A4**	6.407	0.0123	0.914	604.5	7.98	0.917
**B1**	6.624	0.0121	0.963	751.7	9.76	0.964
**B2**	6.595	0.0082	0.993	731.1	6.26	0.993
**B3**	6.502	0.0086	0.985	655.4	6.00	0.985
**B4**	6.414	0.0133	0.910	609.3	9.08	0.906
**C1**	6.672	0.0090	0.971	769.0	7.53	0.972
**C2**	6.586	0.0141	0.964	723.0	11.09	0.962
**C3**	6.540	0.0118	0.962	690.5	8.72	0.961
**C4**	6.358	0.0115	0.908	573.3	9.91	0.916
**D1**	6.676	0.0189	0.953	788.5	16.74	0.953
**D2**	6.707	0.0166	0.910	814.7	14.93	0.908
**D3**	6.724	0.0163	0.951	828.3	14.95	0.954
**D4**	6.730	0.0161	0.946	833.7	14.85	0.949

The calculated a-values and b-values achieved from the double constant equation and Elovich equation fitting curves were consistent with the experimental results, which accurately described the fluorine adsorption processes in biochar-amended soil samples. The fitting results suggested that water-soluble fluorine adsorption in soil samples involved complex processes including adsorption, diffusion, dissolution, mineralization, and their combined effects [38]. The fluorine adsorption capacities ranking for soil samples treated with different types of biochars were as follows: straw biochar (500°C) treated soil > control soil > swine manure biochar (650°C) treated soil > swine manure biochar (500°C) treated soil > swine manure biochar (350°C) treated soil.

### Adsorption isotherms of fluorine

#### Adsorption isotherms of fluorine using biochars

**Fig 3:**
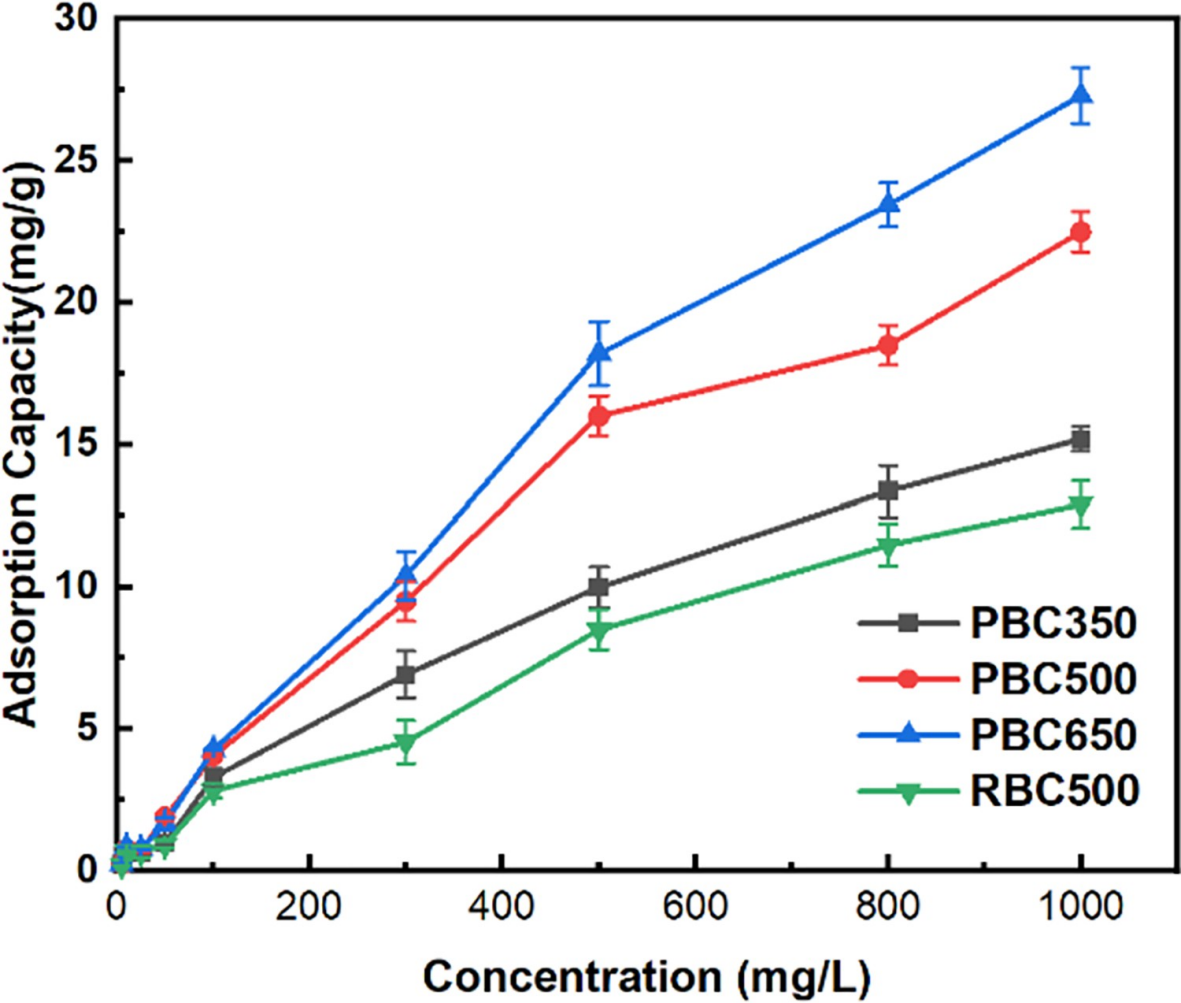
Adsorption isotherms curves on biochars. (Note: weight of biochar = 0.1 g, solution volume = 20 mL, pH = 7, 25°C).

As depicted in Fig 3, the quantities of fluorine adsorbed by biochars at room temperature exhibited an ascending trend with an increased concentrations of fluorine. The fluorine adsorption capacities for all the biochars showed more rapid escalation when the fluorine concentrations range from 10–500 mg/L than when the fluorine concentrations range from 500–1000 mg/L.

The Langmuir isotherm, based on assuming monomolecular layer adsorption, and the Freundlich isotherm, based on assuming multimolecular layer adsorption, were employed for the isothermal adsorption experimental result fitting. The fluorine ion adsorption capacitites increased linearly with fluorine concentration range from 0–800 mg/L. The results of the Langmuir and Freundlich isotherm for the fluorine adsorption were presented in Table 5. The adsorption equilibriums of fluorine by swine manure biochar and straw biochar aligned with the Langmuir and Freundlich models. Notably, the Freundlich isotherm’s 1/n value was less than one, which indicated that a single molecular layer of adsorption primarily dominated the fluorine adsorption using biochars.

**Table 5:** Adsorption isotherm parameters for the fluorine adsorption using biochars.

Samples		Langmuir			Freundlich		
		*q*_*max*_ (mg/g)	B (L/mg)	R^2^	K_f_ ((mg/kg) (L/kg)^1/n^)	1/n	R^2^
**PBC350**		24.252	0.00122	0.992	0.126	0.662	0.985
**PBC500**		43.438	0.00103	0.992	0.160	0.698	0.985
**PBC650**		73.659	0.00057	0.996	0.108	0.789	0.993
**RBC500**		24.899	0.00096	0.993	0.094	0.697	0.986

The following two equations (Eqs (11) and (12)) were used to describe the relationship between the adsorbed fluorine amount Q_e_ and the fluorine concentration C_e_ at the adsorption equilibrium.

$$Langmuir\;isothermal\;adsorption\;equation:\frac{q_{e}}{q_{max}}=\frac{BC_{e}}{1+BC_{e}}$$ (11)

$$Freundlich\;isothermal\;adsorption\;equation:\;q_{e}=K_{f}C_{e}^{\frac{1}{n}}$$ (12)

Where C_e_ (mg/L) represented the remaining fluorine ion concentration in the solution after adsorption equilibrium, B represented the adsorption strength.

Results of the Langmuir isotherm showed that the maximum fluorine adsorption capacity *q*_*max*_ was 73.66 mg/g for swine manure biochar (650°C), which surpassed that of swine manure biochar obtained at 500°C (*q*_*max*_ = 43.44 mg/g), straw biochar obtained at 500°C (*q*_*max*_ = 24.90 mg/g), and swine manure biochar obtained at 350°C (*q*_*max*_ = 24.25 mg/g). These indicated that, under identical feedstock conditions, an increase in pyrolisis temperature for biochar productions would enhance the specific surface areas, thereby improving fluorine adsorption capacities [39].

The fluorine adsorption capabilities for some previously reported adsorbents were compared with that for PBC650 and RBC500, as shown in Table 6. To the best of our knowledge, PBC650 showed more efficient fluorine adsorption capacities than that of any previously studied adsorbents.

**Table 6:** Comparison of fluorine adsorption capacities for PBC650 and RBC500 with for previously reported adsorbents.

Adsorbent	pH	*q*_*max*_ (mg /g)	Adsorption equilibrium time	Reference
**Charcoal**	5.0–5.5	1.42	12 h	19
**Bamboo charcoal**	5.0–5.5	1.65	12 h	19
**Hydrothermal modified fly ash**	7	47.35	1 h	20
**Nitrate acidified activated alumina loaded lanthanum**	6	17.64	2 h	21
**Nitrate acidified activated alumina loaded magnesium**	6	10.96	2 h	21
**Diatomite**	6	32.2	2 h	22
**Calcium bentonite**	6	4.42	3 h	22
**Bamboo charcoal**	6	6.58	4 h	22
**Rice husk biochar**	6	3.42	5 h	22
**Watermelon Rind (Citrullus lanatus) Biochar**	5–7	9.5	3 h	23
**PBC650**	7	73.66	4 h	This work
**RBC500**	7	24.90	4 h	

#### Adsorption isotherms of fluorine in biochar treated soils

**Fig 4:**
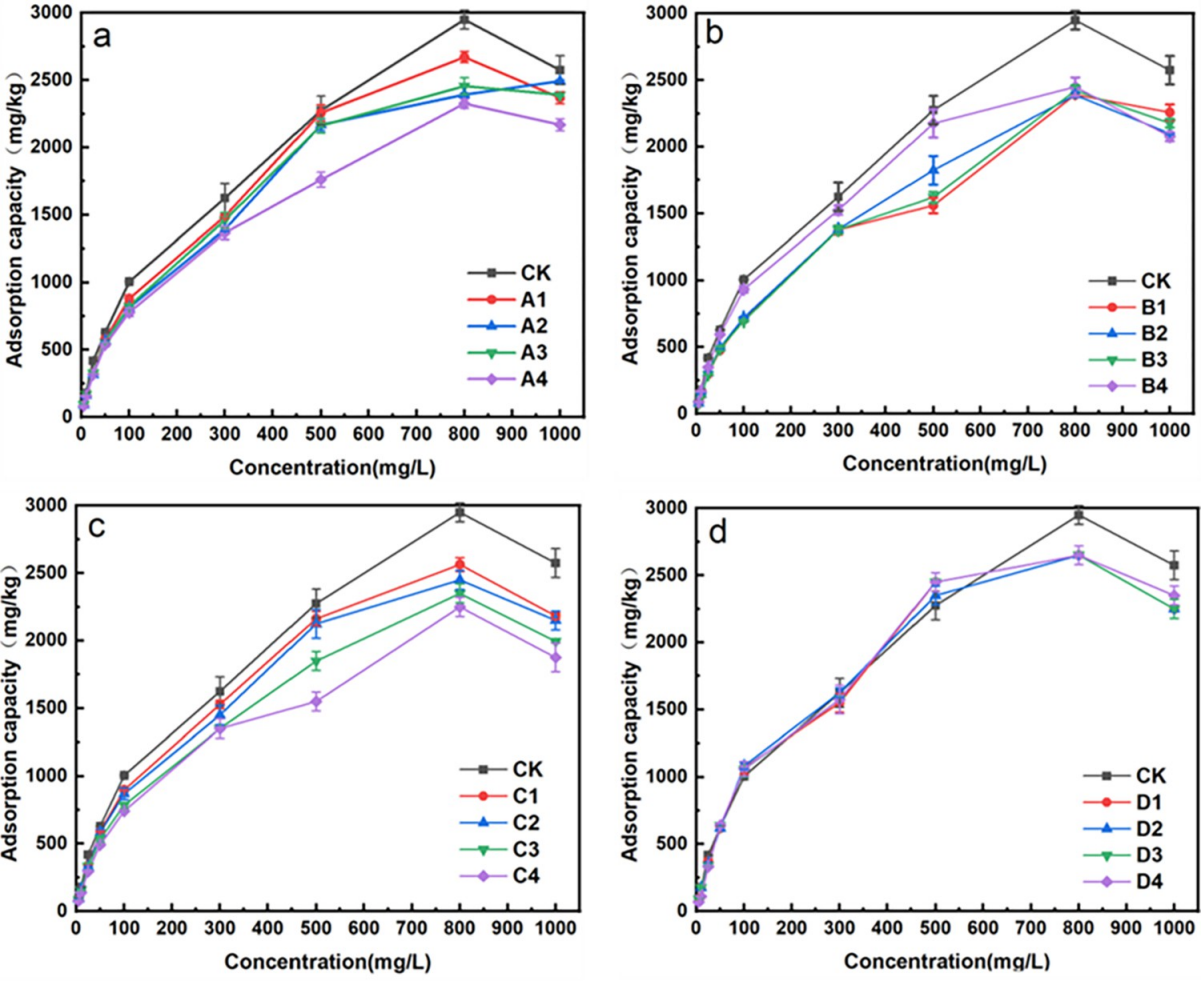
Adsorption isotherms curves in biochar treated soil samples. (Note: weight of soil = 1.0 g, solution volume = 20 mL, pH = 7, 25°C).

Isotherms illustrating fluorine adsorption capacities in different soil samples were presented in Fig 4. The fluorine adsorption capacities for all soil samples increased with the solution fluorine concentrations increased to 800 mg/L. The fluorine adsorption capacities for the soil samples treated with swine manure biochars were all lower than that of the control soil sample. While for the soil samples treated with straw biochars, it showed a little higher fluorine adsorption capacities than that of the control soil with the fluorine concentrations range from 100–500 mg/L.

When fluorine concentrations at 5–50 mg/L and 800–1000 mg/L, the rank for fluorine adsorption capacities was in this order: control soil>RBC500-treated soil>PBC300-treated soil>PBC500-treated soil>PBC650-treated soil. When the initial fluorine concentration was 100–500 mg/L, fluorine adsorption capacities did not show significant differences between RBC500-treated soils and the control soils. Still, both of them showed better fluorine adsorption capacities than that of the soil samples treated with swine manure biochars. The order for fluorine adsorption capacities with the soil samples treated with swine manure biochar was PBC350-treated soil > PBC500-treated soil > PBC650-treated soil. For the biochar prepared at the same pyrolysis temperature, different ratios added to the soil samples did not show obvious differences for fluorine adsorption capacities.

Langmuir and Freundlich isotherm were employed for the isothermal adsorption experimental result fitting with using different biochar-treated soil samples, as shown in Table 7. The R^2^ using Langmuir isotherm exceeded 0.94, and the R^2^ using Freundlich isotherm exceeded 0.90, both showed excellent fitting. Due to the closeness of R^2^ values for Langmuir and Freundlich isotherm, we additionally used chi-square test statistic (Eq 13) [40] for statistical analysis (Table 8). The X^2^ values of Langmuir isotherm were much smaller than that of Freundlich isotherm, which indicated that the Langmuir isotherm models would be better for describing the fluorine adsorption processes.

**Table 7:** Adsorption isotherm parameters for the fluorine adsorption in biochar treated soils.

Sample	Langmuir			Freandlich		
	*q*_*max*_ (mg/g)	B (L/mg)	R^2^	K_f_ ((mg/kg) (L/kg)^1/n^)	1/n	R^2^
**CK**	3548.51	0.0037	0.957	108.507	0.476	0.945
**A1**	354.76	0.0036	0.965	102.638	0.472	0.940
**A2**	3232.89	0.0032	0.982	85.286	0.497	0.974
**A3**	3191.31	0.0034	0.982	93.101	0.482	0.967
**A4**	892.74	0.0033	0.975	83.601	0.482	0.972
**B1**	3150.34	0.0026	0.963	106.614	0.464	0.937
**B2**	3011.31	0.0033	0.968	103.448	0.462	0.942
**B3**	2968.07	0.0028	0.965	97.799	0.467	0.939
**B4**	2782.64	0.0051	0.961	81.650	0.481	0.960
**C1**	2964.56	0.0043	0.967	117.596	0.445	0.935
**C2**	2921.06	0.0041	0.971	110.638	0.451	0.939
**C3**	2777.11	0.0039	0.956	96.997	0.460	0.931
**C4**	2712.63	0.0036	0.961	84.469	0.475	0.949
**D1**	3443.80	0.0049	0.940	143.641	0.424	0.911
**D2**	3358.10	0.0054	0.956	153.194	0.413	0.904
**D3**	3226.86	0.0050	0.949	145.061	0.422	0.901
**D4**	3117.34	0.0048	0.949	137.036	0.433	0.914

**Table 8:** X^2^ statistics of two models.

Biochar	Langmuir	Freandlich	Biochar	Langmuir	Freandlich
**CK**	155	355			
**A1**	111	320	**C1**	94	387
**A2**	122	201	**C2**	99	312
**A3**	89	199	**C3**	152	244
**A4**	112	118	**C4**	127	230
**B1**	129	171	**D1**	144	559
**B2**	108	229	**D2**	97	606
**B3**	112	190	**D3**	132	587
**B4**	94	569	**D4**	119	942


$$X^{2}=\sum_{N}^{i=1}\frac{{\left(q_{exp,i}-q_{m,i}\right)}^{2}}{q_{m,i}}$$ (13)

Where *q*_*exp*,*i*_ and *q*_*m*,*i*_ represented the equilibrium fluorine adsorption capacities (mg/g) obtained from the experiments and models, respectively. N meant the sample size.

In the Langmuir isotherm fitting data, the maximum fluorine adsorption capacities ranked as follows: control soil > RBC500-treated soil > PBC350-treated soil > PBC500-treated soil > PBC650-treated soil. For the biochar prepared at the same pyrolysis temperature, the higher the proportions of biochars added, the lower the fluorine adsorption capacities for biochar-treated soil samples, in which the soil sample treated with 2% PBC650 exhibited the lowest fluorine adsorption capacity.

### Desorption isotherms

The quantities of soil fluorine desorption rose with high initial liquid fluorine concentrations for diverse biochar treatment soil samples. The amounts and rates of fluorine desorption for the four group samples exceeded that of the control soil when the initial fluorine concentration ranged from 10 to 500 mg/L, which indicated that the addition of biochars promoted the specialized fluorine adsorption by the soils [41]. Among the four group samples, the lowest desorption capacity was found in RBC500-treated soils, which had the strongest ability to sequester fluorine. The isothermal desorption process results were fitted using the Freundlich equation (Table 9), and large K_f_ values obtained from the fit curves indicated weak desorption capacities. The K_f_ values of all four group samples were larger than those of the control soil, and it increased with the increased ratios of biochars applied. The results suggested that the applications of biochars had an inhibitory effect on the desorption of fluorine in the soils.

**Table 9:** Desorption isotherm parameters for the fluorine desorption in different soil samples.

Freandlich							
Sample	K_f_ ((mg/kg) (L/kg)^1/n^)	1/n	R^2^	Sample	K_f_ ((mg/kg) (L/kg)^1/n^)	1/n	R^2^
**CK**	0.029	0.396	0.943				
**A1**	0.036	0.397	0.872	**C1**	0.055	0.318	0.886
**A2**	0.051	0.340	0.918	**C2**	0.048	0.34	0.843
**A3**	0.051	0.359	0.883	**C3**	0.048	0.356	0.853
**A4**	0.068	0.308	0.847	**C4**	0.06	0.329	0.901
**B1**	0.084	0.243	0.901	**D1**	0.063	0.279	0.924
**B2**	0.082	0.373	0.854	**D2**	0.056	0.306	0.895
**B3**	0.084	0.274	0.929	**D3**	0.041	0.361	0.944
**B4**	0.086	0.237	0.917	**D4**	0.057	0.307	0.943

### Effect of pH on the fluorine adsorption

Soil pH significantly affected the fluorine adsorption efficiencies. It is reported that fluorine adsorption capacities decreased with the increasing pH [19, 42, 43]. The pHs of A4, B4, C4, and D4 samples were 4.41, 5.28, 5.57, and 3.88, respectively, which were 0.81, 1.68, 1.97, and 0.28 higher than the pH of the control soil solution. The pH values of the soil samples increased gradually with the increased ratios of biochars. The pH of the solutions significantly impacted the adsorption processes for biochars due to its effect on the ionization degrees, ion forms, and surface charges of the biochars. Consequently, the solution pH also influenced the fluorine adsorption characteristics using the biochars [44]. The changes of solution pH would lead to the arrangement of the adsorbent surface charges, which influenced the number of adsorption sites, the structural properties of functional groups on the biochar surfaces, and the processes of mineral component dissolutions in the solutions [45].

**Fig 5:**
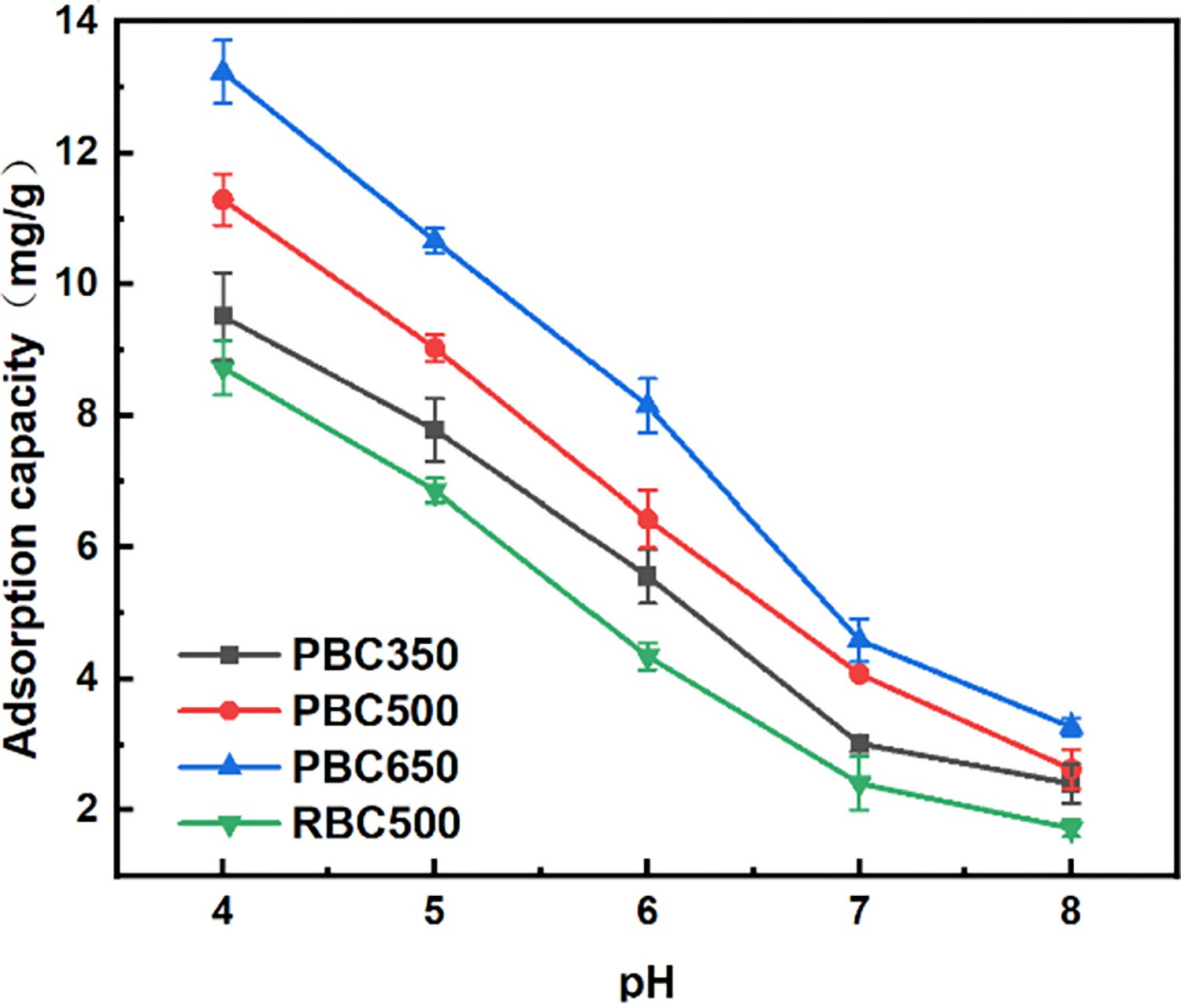
Adsorption curves using different biochars under different pH.

**Fig 6:**
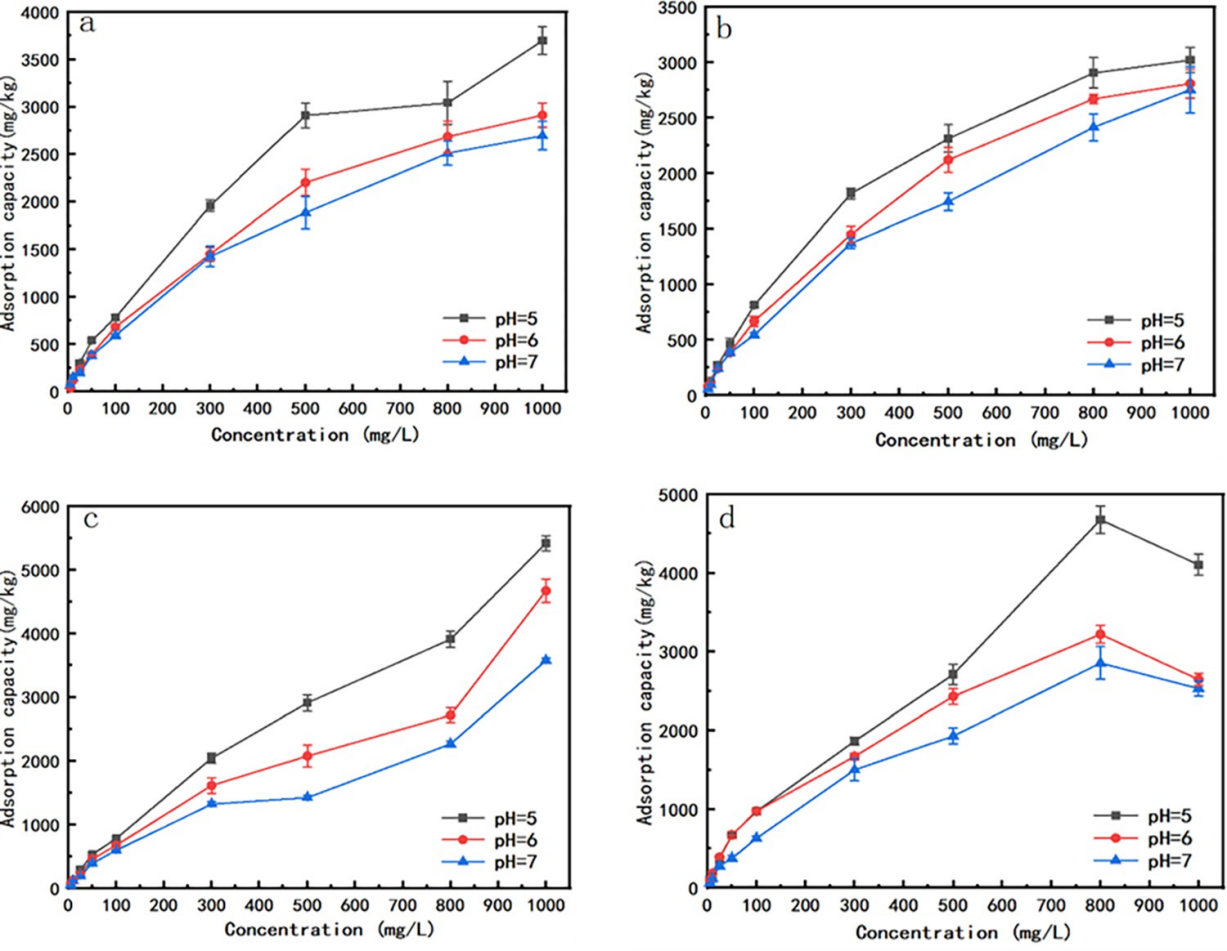
Soil adsorption curves under different pH. (Note: a, PBC350-treated soil; b, PBC500-treated soil; c, PBC650-treated soil; d, RBC500-treated soil).

The results of the fluorine adsorption capacities for biochars under different pH were shown in Fig 5. The findings indicated that the fluorine adsorption capacities of all four types of biochars gradually decreased with the increasing pH. These results were consistently confirmed by the fluorine adsorption experiments for different soil samples conducted at various pH, as shown in Fig 6. Furthermore, with increasing pH, the fluorine adsorption efficiencies decreased for soil samples treated with both low and high concentrations of biochars. Under high pH conditions of soils, fluorine anions adsorbed on the exchangeable positive charges of clay particles, organic matter particles, and hydrated oxides would be exchanged with dissociative OH^-^ existed at high pH, which resulted to the release of fluorine anions, followed by the reduced fluorine adsorption capacities for soil samples and the increased fluorine concentrations in the soil solutions [46].

### Effect of interfering ions on the adsorption

**Fig 7:**
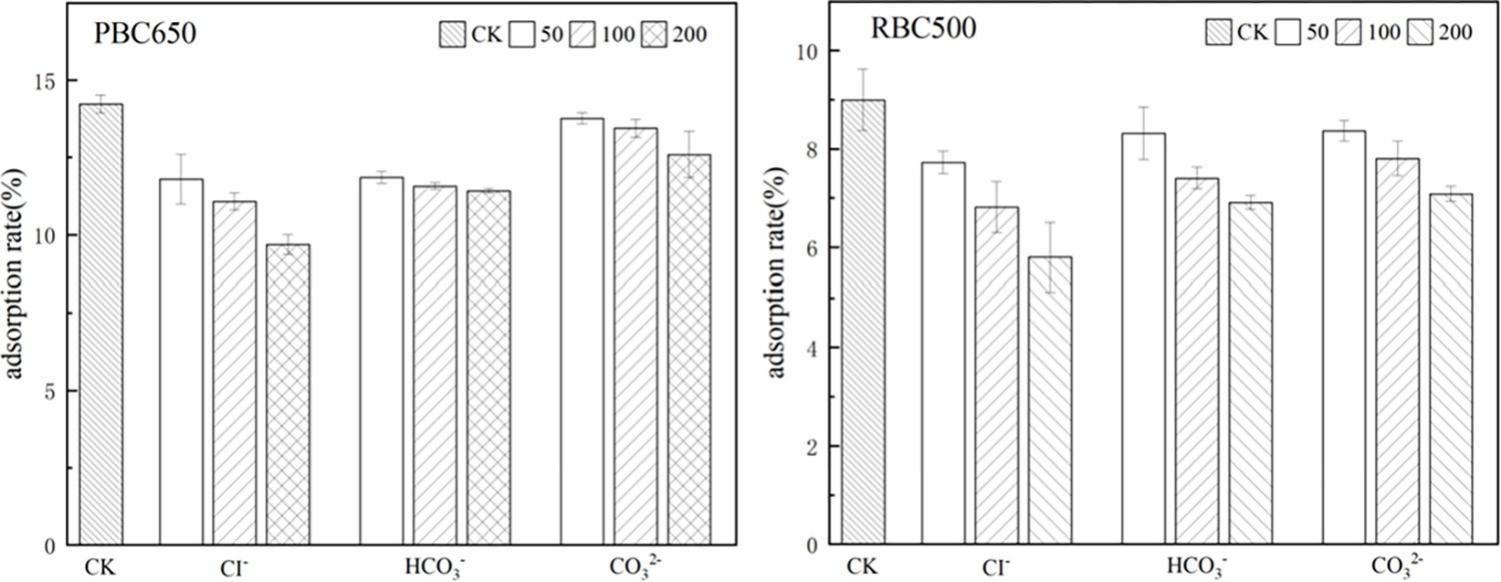
Effect of interfering ions at different concentrations on fluoride adsorption. (Note: weight of biochar = 0.1 g, solution volume = 20 mL, CK is 50 mg/L of F^-^, interfering ions concentration = 50 mg/L、100 mg/L and 200 mg/L, pH = 7, 25°C).

The presence of interfering ions does affect the fluoride removal capacity of the adsorbent. Therefore, we selected three common anions (Cl^-^、HCO_3_^−^、CO_3_^2−^) and two types of biochar (PBC650、RBC500)to study successively. Each anion was set 50、100 and 200 mg/L, CK is 50 mg/L of F^-^. As Fig 7, the influence of anions on the biochar is Cl^-^ >HCO_3_^−^ >CO_3_^2−^. Higher concentrations of interfering ions have a greater effect on biochar adsorption. This is attributed to the competitive adsorption of anions with fluoride ions on the adsorbent surface. This is attributed to the competitive adsorption of anions with fluoride on the adsorbent surface. The biochar has a finite number of adsorption sites. When the anion concentration is high, the probability of anion reaching the adsorption site is greater, and the fluoride removal rate decreases.

### Characterisations of biochars

**Fig 8:**
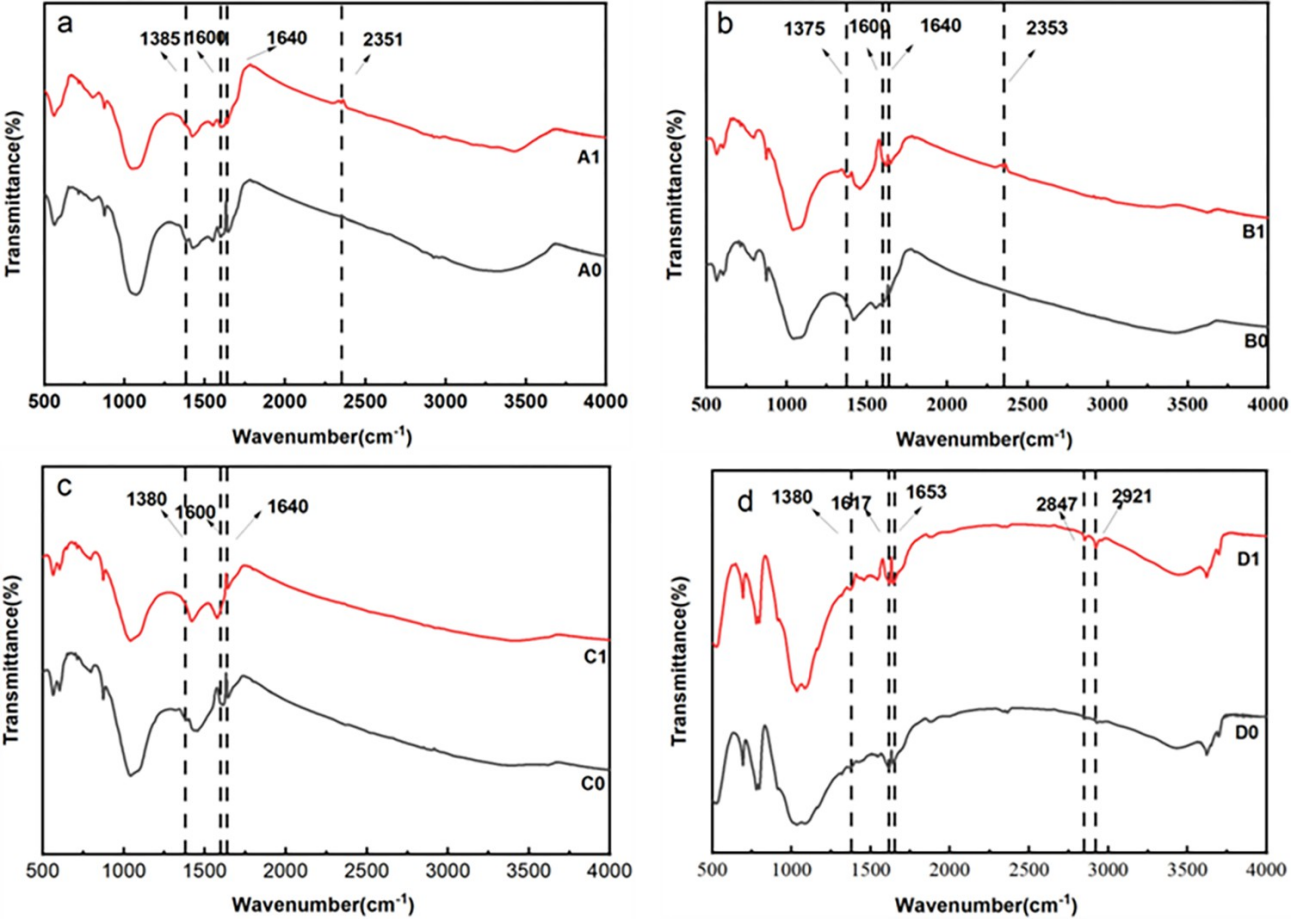
Infrared spectra before and after biochar adsorption. (Note: a, PBC350-treated soil; b, PBC500-treated soil; c, PBC650-treated soil; d, RBC500-treated soil).

Fourier transform infrared (FTIR) spectra was used for the measurements of the mixture structures before and after the adsorption of fluorine by four types of biochars, as shown in Fig 8. The positions of the functional group absorption peaks for the three kinds of swine manure biochars prepared at different pyrolysis temperatures were consistent. After the fluorine adsorption occurred, notable changes were observed. For instance, with certain incubation time of fluorine solutions for A1 sample, the intensity of the -CH_3_ vibration absorption peak at 1385 cm^-1^ weakened, and the telescopic vibration peaks of -COOH (1600–1640 cm^-1^) were attenuated. The peak at 2351 cm^-1^, representing the telescopic vibration of the triple bonds and accumulating double bonds, showed increased intensities. Similar trends were observed for B1 and C1 samples, with specific alterations in vibrational peaks corresponding to -CH_3_, -COOH, and carbon-carbon or carbon-nitrogen triple bonds. D1 sample displayed strengthened vibrational peaks of the conjugated C = O group (1575–1631 cm^-1^) and the -C-H vibration peaks (2847–2921 cm^-1^) after certain incubation time for fluorine adsorption.

The FTIR spectra measurements of different biochars for the fluorine adsorptions indicated significant changes in the absorption bands at 1385 cm^-1^, 1600–1640 cm^-1^, and 2351 cm^-1^, which were corresponding to C-H expansion and contraction vibrations, -COOH, and carbon-carbon or carbon-nitrogen triple bonds, respectively. These changes suggested that fluorine was adsorbed by biochar active sites, in which -COOH group was identified as key adsorption sites for fluorine.

**Fig 9:**
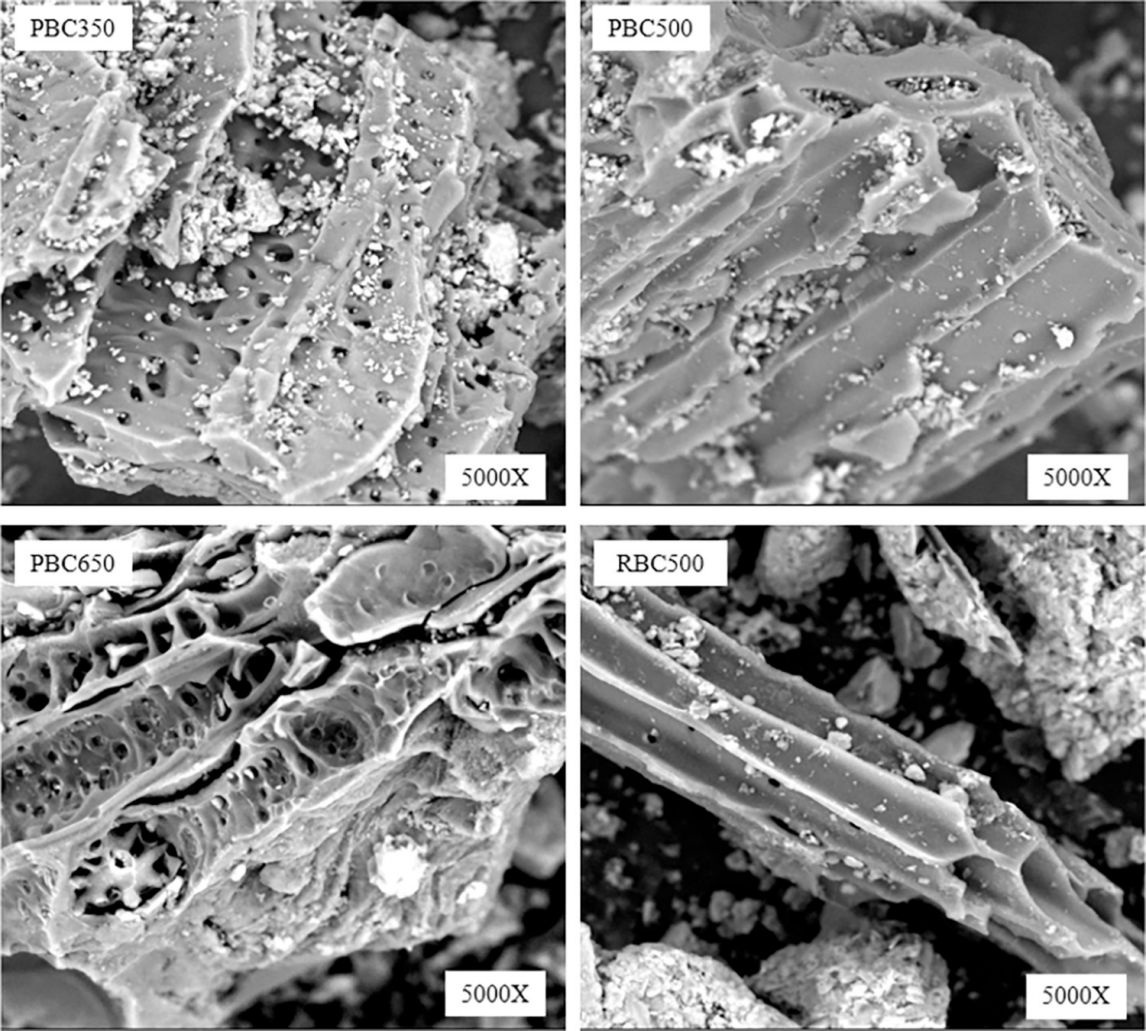
SEM images of different types of biochars.

In the biochar adsorption experiments, solution pH using four types of biochars were uniformly adjusted to pH 7. With the SEM measurement shown in Fig 9, it was found that higher pyrolysis temperatures for biochar preparations resulted in more pore holes and thicker pore walls of biochars. The increases of specific surface areas and decreases of mean pore diameters enhanced the biochar capacities for fluorine adsorptions. The fluorine adsorption experiments using soil samples indicated that the mixture pH significantly influenced fluorine adsorption, surpassing the impact of other factors. The additions of biochars to the soils led to increased pH, which in verse resulted to decreased fluorine adsorption capacities. At the same time, it was found that the higher the amounts of biochars added, the higher the soil pH, and the lower the fluorine adsorption capacities. Therefore, pH emerged as a critical factor affecting fluorine adsorption capacities for soil samples.

Overall, this study still had limitations as only fresh biochars obtained by thermal pyrolysis were used and the presences of other interfering ions (HPO_4_^2−^、PO_4_^3−^、SO_4_^2−^、NO_3_^–^ and metal cation) that could affect the fluoride removal capacity of the adsorbent. Huang et al. [47] found that the surface pore structures of biochars would collapse after naturally aging for three years in the field. With the increased field aging time, the BET-specific surface areas, total pore volumes, mesoporous pore volumes and average pore sizes of biochar increased, while the micropore surface areas decreased. Thus, the aged biochars often appeared with different characteristics might change their capabilities in adsorbing pollutants and their effects on the fluorine adsorption capacities by mixing with the soils. Duan et al. [48] found that the higher the charge radius ratio of anion, the stronger the interactions between the adsorbent and anions, which would inhibit the fluoride adsorption capacities of adsorbent in solutions. Thus, it was also necessary to study the effects of aged biochars and interfering ions on fluoride adsorption capacities in the following studies.

## Conclusions

In this comprehensive study, the impacts of swine manure and straw biochars on fluorine adsorption-desorption processes in soils were thoroughly studied through indoor batch equilibrium adsorption tests. The introduction of biochars to the soils triggered discernible shifts in the adsorption dynamics of fluorine. The adsorption of fluorine using swine manure and straw biochars-treated soil samples achieved equilibrium at approximately 4 hours of incubation. The adsorption kinetics of fluorine using swine manure and straw biochars were in accord with quasi-two-stage kinetic model. Furthermore, the adsorption kinetics of fluorine on biochars-treated soil samples conformed to both of the double constant equation and Elovich equation. By applying Langmuir and Freundlich isothermal adsorption models, the fluorine adsorption processes were confirmed to be dominated by monomolecular layer adsorption. Interestingly, the fluorine adsorption performances for biochars-treated soil samples displayed diminishing trends with increased proportions of biochar added. Concurrently, the fluorine adsorption capacities using biochars gradually decreased with the increased pH. The observed decreased fluorine adsorption capacities for biochars-treated soil samples were markedly associated with increased soil pH after adding biochars. In conclusion, this study provides valuable insights into the intricate interplay between biochars, soil properties, and fluorine adsorption dynamics. The findings indicated that multifaceted processes occurred during soil remediation, and pH would be deemed as a critical factor that affected the efficacies of biochars on ion adsorption-desorption capacities for soils.
